# Gender differences in psychosomatic complaints across occupations and time from 2006 to 2018 in Germany: a repeated cross-sectional study

**DOI:** 10.1186/s12889-025-21462-8

**Published:** 2025-02-01

**Authors:** Julia Grasshoff, Batoul Safieddine, Stefanie Sperlich, Johannes Beller

**Affiliations:** Department for Medical Sociology, Hannover Medical School, Hanover, Germany

**Keywords:** Gender differences, Mental health, Occupational health, Psychosomatic complaints, Trends, Collar

## Abstract

**Background:**

Previous research indicates that women report more psychosomatic complaints at work compared to men. However, there is a lack of research examining this gender gap across different occupational subgroups and over time.

**Methods:**

The study utilized data from the nationwide German Employment Survey of the Working Population on Qualification and Working Conditions conducted in 2005/2006, 2011/2012, and 2017/ 2018. First, gender differences in psychosomatic complaints were analysed within the occupational subgroups categorized as white-collar high-skilled, white-collar low-skilled, blue-collar high-skilled and blue-collar low-skilled workers. Second, gender stratified time trends of psychosomatic complaints were analysed. A total of 58,759 participants were included in the analysis.

**Results:**

Women consistently reported significantly higher levels of psychosomatic complaints compared to men across all years examined. The largest differences were observed in white-collar high-skilled occupations. From 2005/2006 to 2011/2012, gender differences increased; from 2011/2012 to 2017/2018, they stagnated.

**Conclusions:**

The study revealed that women experience more psychosomatic distress at work than men in all occupational subgroups and time points. White-collar high-skilled workers showed the highest gender gap in psychosomatic complaints. The gender gap widened from 2005/2006 to 2011/2012 and remained stable from 2011/2012 to 2017/2018. Future research should investigate the reasons and implications of this phenomenon, especially considering the increasing proportion of high-skilled white-collar workers, where the gender gap is most evident.

**Supplementary Information:**

The online version contains supplementary material available at 10.1186/s12889-025-21462-8.

## Background

Psychosomatic complaints are physical symptoms that are influenced by psychological factors, such as stress, emotions, or personality traits. They are prevalent among workers and can negatively impact their well-being, productivity, and overall quality of life. Moreover, psychosomatic complaints have been demonstrated to predict work absences and disability [1, 2].

### Gender and occupational differences in psychosomatic complaints at work

Working conditions can have a profound impact on mental health. In a meta-analysis, Seidler et al. [3] reviewed six cohort studies and identified psychosocial working conditions—such as high workload, high quantitative, mental, or emotional demands, and low social support—as significant predictors of burnout, with emotional exhaustion being its most prominent component. Similarly, Aronsson et al. [4] conducted a meta-analysis and found strong associations between higher job control and reduced emotional exhaustion, as well as between low workplace support and increased emotional exhaustion. Additionally, their findings highlighted links between emotional exhaustion and factors such as workplace justice, high demands, heavy workload, low rewards, insufficient supervisor or co-worker support, and job insecurity.

At the same time, occupational gender inequalities in psychosomatic complaints continue to be evident in research. Women consistently report higher levels of psychosomatic complaints at work compared to men [6–10]. Additionally, women tend to have higher rates of absenteeism [11, 12] and seek medical attention more frequently, particularly for non-specific symptoms such as headaches and fatigue [12, 13]. However, findings regarding gender differences in norms and attitudes towards sick leave are mixed. Some studies suggest no gender difference in perceptions of the appropriateness of sick leave for a given level of illness [14], while others indicate that men are less likely to perceive a certain level of illness as warranting sick leave [15]. Interestingly, one study found a stronger association between depressive symptoms and sick leave among men compared to women, although the variability in study outcomes was greater for men [16].

Occupational differences in psychosomatic complaints present a complex pattern. Myrtek et al. [17] reported mixed findings: while white-collar workers showed higher stress levels after work, blue-collar workers experienced greater mental strain during work hours. Research by Hämmig & Bauer [18] revealed that blue-collar low-skilled workers exhibited lower stress and burnout levels compared to their white-collar high-skilled counterparts, yet simultaneously demonstrated poorer self-rated health, reduced physical functioning, and increased sickness absence. The authors postulate a reverse social gradient in mental health outcomes. In a study of Finnish employees, Lahti et al. [5] observed higher levels of emotional exhaustion among managers and professionals, with manual workers reporting lower levels. This suggests elevated distress among white-collar high-skilled workers, though job demands moderated these differences.

Regarding gender disparities within occupational groups, Grasshoff et al. [19] analyzed the 2018 wave of the three German samples examined in this manuscript. Using similar stratification by blue-collar versus white-collar employment and skill level, they identified varying gender differences in psychosomatic complaints across occupational subgroups. Women in white-collar positions, especially those in high-skilled roles, reported more frequent psychosomatic complaints than their male colleagues. However, the temporal stability of this gender gap and potential trends over time remain unexplored.

### Modern work: more women, more white-collar, more qualification

The world of work has changed significantly over the past few decades. Recently, there has been a notable increase in female representation within the workforce, while the proportion of men employed has remained relatively stable. For example, between 1998 and 2021, the cumulative growth in employment across European countries has disproportionately favoured women, with a ratio of 2:1 [20–23]. Several factors contribute to this rise in female employment. Although the gender pay gap is still present, wages for women increased and the gap is declining [24]. Additionally, with women still taking a bigger part in care work than men [25] due to societal pressures, the increased availability of childcare facilities plays an important role. Between 2013 and 2023, the number of children cared for in daycare centres in Germany increased by 22% and the number of children under the age of three even by 43% [26]. However, the provision of childcare in Germany exhibits significant regional differences, particularly a pronounced East–West divide. In 2022, 40.8% of children under the age of three in the former East German states received full-day care, compared to only 14.8% in the former West German states. This disparity persists when examining childcare for children aged three to six years, with 73.4% of children in the East receiving full-day care, compared to just 41.4% in the West [27]. With regard to the disproportionate care work undertaken by women, this regional disparity particularly affects the burden placed on women. Moreover, policy changes such as the introduction of the Federal Parental Allowance and Parental Leave Act in 2007 have resulted in an increase in fathers taking parental leave. This shift has the potential to alleviate the burden traditionally placed on women. However, women remain dependent on their partner's decision and may face challenges arising from conflicting expectations within their partner’s workplace. Additionally, the duration of women's education has increased and, in recent years, has even surpassed that of men [28]. At the same time, the proportion of women working full-time has remained relatively unchanged in recent years compared to men [21, 22], which stabilizes gender inequalities in financial security and independence throughout both working life and retirement.

A significant transformation has occurred in workforce sectors, job demands, and occupational stressors. Contemporary work increasingly emphasizes high-skilled and white-collar tasks, driven by advances in information and communications technology and the growth of cognitive tasks requiring advanced education, specialized skills, and expertise [29]. Concurrently, there has been a reduction in repetitive and physically demanding tasks typically associated with blue-collar work [29]. A distinct emerging sector is gig work, often comprising blue-collar low-skilled tasks such as passenger transport and food delivery. An EU-wide online survey by Urzì and Férnandéz-Macías [30] documented its growth from 10.1% in 2017 to 12.3% in 2018, measuring participation as main, secondary, or marginal income source. German respondents predominantly reported using gig work as supplementary income, potentially escaping detection in surveys focused on primary occupations. Educational attainment has risen significantly, with more individuals completing high school and university education before entering the workforce [28, 31], many gravitating toward the expanding sector of high-skilled white-collar positions [29]. This shift has transformed work-related stressors. While blue-collar jobs traditionally involved physical strains such as chemical exposure and heavy lifting [12, 32]—concerns that shaped early occupational health monitoring and data collection [33]—white-collar occupations typically entail heightened psychosocial and emotional demands [32–34].

### What are time trends of gender differences in psychosomatic complaints at work?

Despite the marked changes in the world of work, studies are lacking that examine whether the gender gap in psychosomatic symptoms at work has also changed over time. Women experience more psychosocial work demands across different collars and levels of qualification compared to their male colleagues [10]. At the same time, there is evidence suggesting that gender differences in psychosomatic complaints are most pronounced in white-collar high-skilled occupations [19], where a growing demand for workers has also been observed. It is plausible to anticipate an escalation in gender disparities over time across the entire workforce. We pose the questions: “Has the gender gap in psychosomatic complaints changed over time?” and in particular “Is the white-collar high-skilled occupation group over time consistently the group with the biggest gender differences in psychosomatic complaints?” Based on the literature it is to be expected that the proportion of white-collar high-skilled jobs is increasing and that the biggest gender differences will be found in this subgroup. Therefore, an increase of gender differences in the populations over time is expected.

## Methods

Three samples from three waves of the nationwide German Employment Survey of the Working Population on Qualification and Working Conditions conducted in 2005/2006, 2011/2012, and 2017/2018 were used. For ease of reference, these survey waves will be denoted as 2006, 2012, and 2018. The survey was conducted by the Federal Institute for Vocational Education and Training (BIBB) in collaboration with the Federal Institute for Occupational Safety and Health (BAuA). Ethics approval was deemed unnecessary as this study involved secondary analysis of pre-existing data that had been previously collected and published. Across all waves, participants were randomly selected from the national telephone registry using their landline phone numbers. In the 2018 wave, an additional 30% of the sample was contacted via mobile phone to address the increasing prevalence of individuals without landline phones. All survey participants provided informed consent. Data were collected from individuals aged at least 15 years old who were employed for a minimum of 10 h per week in Germany. Individuals experiencing work interruptions (e.g., due to maternity leave or illness) of up to three months were included. Interviews were conducted one-on-one using structured questionnaires.

The questionnaire used to gather the dataset was not developed specifically for the current study. The authors did not develop or own the questionnaire but are only users. The questionnaire was used to gather the data which was done by the owner. The owner of both the questionnaire and the data is the Bundesinstitut für Berufsbildung Deutschland [Federal Institute for Vocational Education and Training Germany] (BIBB). The dataset and questionnaire are freely available for academic purposes. They are available on request through a public repository after having signed an agreement with the owner (https://www.bibb.de/de/1403.php). SUFs are distributed directly via the BIBB-FDZ. For this purpose, an application form (available online via https://www.bibb.de/dokumente/pdf/BIBB_FDZ_Antrag_SUF_FDZ_deutsch.pdf) must be completed, signed and sent to the BIBB-FDZ by post (BIBB—Bundesinstitut fuer Berufsbildung Arbeitsbereich 1.5: Forschungsdatenzentrum, Postfach 20 12 64, 53,142 Bonn, Germany) or e-mail (fdz@bibb.de). Data access and permission to use has been granted to the researchers Johannes Beller and Julia Grasshoff.

Data were accessed for research purposes by the corresponding author on 2nd of May 2023. Authors did not have access to information that could identify individual participants.

The study used weights, as provided by the data, to account for sampling design effects and potential response bias. These weights were calculated based on the German Microcensus data, incorporating adjustments for household selection probability, regional distribution, and sociodemographic characteristics. The weighting procedure ensured representativeness of the sample with respect to federal state, urbanization, household size, occupational status, and key demographic variables. However, similar results were obtained when using unweighted analyses [35, 36]. The original dataset included 60,048 cases. After deleting 1072 cases with missing values listwise (1.79%), 58,976 cases remained for the analysis. Most cases were omitted due to missing values in the variables “occupation” (293 cases) and “psychosomatic complaints” (291 cases).

### Demographic and sociodemographic measures

Variables used in the analysis were gender, age, working hours, parental status, and occupation. Gender was treated as a binary nominal variable (“male”, “female”). Age was treated as a continuous metric variable. Working hours was treated as binary nominal variable (“full-time” meaning 36 h or more, “part-time” meaning less than 36 h). It was included as a covariate due to the finding that long working hours (more than 35 h/week) are associated with diminished well-being, although the literature is not entirely conclusive [37, 38]. Parental status was treated as a binary nominal variable (“yes, “no”). It was included as a covariate due to findings that balancing work and family can increase rates of chronic illness and poorer self-rated health [39]. The self-selected occupation of the participants was classified by the data owners using the ISCO88 one-digit coding. The 10 major groups in the data set were the following: Code 1 included legislators, senior officials, managers, code 2 included professionals, code 3 included technicians and associate professionals, code 4 included clerks, code 5 included service workers, shop and market sales workers, code 6 included skilled agricultural and fishery workers, code 7 included craft and related trades workers, code 8 included plant and machine operators, code 9 included assemblers and elementary occupations and code 0 included armed forces. These codes were summarized into four comprehensive occupational groups regarding skill level (complexity and range of tasks) and skill specialisation (field of work, tools and kind of goods or services) [40]. The sample size necessitates grouping for the analyses. A grouping as detailed as individual occupations would be too granular; with 10 occupational groups and 2 gender categories, this would result in 20 comparisons each year. The use of 4 occupational groups represents an approach to differentiate the sample while aligning with the background of previous research, ensuring that the creation of excessively small subgroups is avoided. The following four groups were differentiated: white-collar high-skilled occupations (Code 1–3), white-collar low-skilled occupations (code 4 and 5), blue-collar high-skilled occupations (code 6 and 7) and blue-collar low-skilled occupations (code 8 and 9). Armed forces (Code 0) were excluded from the analysis. This is a coding standard used in research before [18, 19] and is also used by the European Foundation for the Improvement of Living and Working Conditions (Eurofound) in their surveys who regularly examine trends in work [41]. Additionally, it takes into consideration the shift in sectors of work as described above [29, 30]. Occupational group was treated as a nominal variable in the analyses.

### Psychosomatic measures

Across all survey years, participants responded to 23 health-related symptoms. Of these, only 13 items maintained consistent presentation across survey waves. The remaining items varied in their general appearance, wording, or structure, with some years combining symptoms that were presented separately in others. These inconsistencies precluded their inclusion in time trend analyses due to compromised comparability. From the 13 consistent symptoms, we selected five items that most clearly reflected psychological manifestations, aligning with our research focus. For completeness, sensitivity analyses incorporating all 13 consistent variables are presented in the Appendix (table A4 and table A5). Regarding psychosomatic complaints, in 2006, participants were asked: “Tell me, if the following health complaints appeared at or immediately after work”; in 2012 and 2018, participants were asked “Tell me, if the following health complaints appeared at work or on workdays. We are interested in complaints that appeared frequently.” The question was followed by offering singular symptoms. The participants could answer “yes” or “no” for each symptom. The offered symptoms were the following: headaches, insomnia at night, tiredness, irritability, and depressed mood. A psychosomatic complaint score was calculated by treating the responses per question as binary variables (yes = 1, no = 0) and adding up the “yes” responses resulting in a range of 0 to 5 points with 0 being the lowest level of reported complaints.

## Data analyses

First, descriptive analyses were calculated in terms of absolute and relative frequencies for each year of the sample.

Given our use of a non-validated psychosomatic complaint score, we conducted both exploratory and confirmatory factor analyses to assess its psychometric properties. The principal-component factor analysis employed the Kaiser criterion, which retains only factors with eigenvalues greater than or equal to 1. Additionally, Cronbach`s Alpha and McDonald’s Omega were calculated as well as Horn’s parallel analysis for principal component analysis.

The repeated calculation of mean differences in psychosomatic complaints for each wave was done via t-tests. With three waves and four collar plus an overall wave value, this resulted in 3 × 5 = 15 t-tests. The significance of mean differences between genders was additionally tested through weighted linear regression for each year to control for covariates, stratifying for each occupational subgroup plus an overall value over all occupational subgroups using an α-level of 5% for each subgroup. Gender was used as independent variable; psychosomatic complaint score was used as dependent variable. Age, parental status and working hours were used as covariates. Effect sizes for the same groups were calculated using Cohen’s d [42]. Furthermore, we first estimated a weighted regression model without interaction effects to estimate direct effects of gender and wave after controlling for the covariates age, parental status, collar and working hours. Then, we estimated a weighted interaction regression model to test if the gender differences did change over time. A weighted interaction model using linear regression was conducted to examine the interaction of gender (categorial variable with two conditions) and wave (categorical variable with three conditions) on psychosomatic complaint score. Age, parental status, collar and working hours were used as covariates. All analysis were performed using StataMP 15.

## Results

### Basic characteristics of the study sample

As displayed in Table 1, of all participants in wave 2006, 48.61% were female; in wave 2012 52.53% were female and in wave 2018 49.76% were female. As seen in Table A1 in the Appendix, of all participants in wave 2006, mean age was 41.31 (*SD* = 10.46) years, in wave 2012 mean age was 46.06 (*SD* = 10.70) years and in wave 2018 mean age was 47.22 (*SD* = 11.31) years. The proportion of white-collar high-skilled workers in the samples grew from 53.13% in wave 2006 to 55.07% in wave 2012 and to 63.97% in wave 2018. The proportion of white-collar low-skilled workers developed from 21.18% in wave 2006 to 21.37% in wave 2012 to 17.05% in wave 2018. The proportion of blue-collar high-skilled workers decreased from 14.27% in wave 2006 to 12.74% in wave 2012 to 9.78% in wave 2018. The proportion of blue-collar low-skilled workers decreased from 11.42% in wave 2006 to 10.82% in wave 2012 to 9.20% in wave 2018. As displayed in Table 1, the occupational subgroup of high-skilled white-collar workers in each year was nearly divided equally in men and women. White-collar low-skilled and blue-collar low-skilled jobs were female-dominated, blue-collar high-skilled jobs were male-dominated.

**Table 1:** Description of the study sample

Survey wave	**2006**		**2012**		**2018**	
Gender	Male	Female	Male	Female	Male	Female
Sample size N/ %	10,187/51.39%	9637/48.61%	9298/47.47%	10,288/52.53%	9829/50.24%	9737/49.76%
Occupational groups, %,						
White-collar high-skilled	49.30%	50.70%	44.89%	55.11%	47.98%	52.02%
White-collar low-skilled	26.55%	73.45%	24.78%	75.22%	27.61%	72.39%
Blue-collar high-skilled	85.86%	14.14%	83.01%	16.99%	84.74%	15.26%
Blue-collar low-skilled	64.09%	35.91%	63.57%	36.43%	71.18%	28.82%

Multiple psychometric methods confirmed that our psychosomatic symptoms form a single underlying factor of psychosomatic symptom burden. First, in accordance with the Kaiser criterion, the single factor had an eigenvalue of 2.42, while all other factors had eigenvalues below 1. This primary factor explained 48.46% of the total variance. Both Horn's parallel analysis and the scree plot also supported the one-factor solution, as seen in Figure AF1 and Figure AF2 in the Appendix. The scale showed satisfactory internal consistency with both Cronbach's Alpha = 0.73 and McDonald's Omega = 0.73. A confirmatory factor analysis of this one factor solution using robust diagonally weighted least squares also demonstrated good model fit (robust CFI = 0.98). The items showed strong factor loadings ranging from 0.56 ("headache") to 0.85 ("depressed mood”), supporting the psychometric validity.

### Gender differences in psychosomatic symptoms stratified by occupational groups

**Fig. 1:**
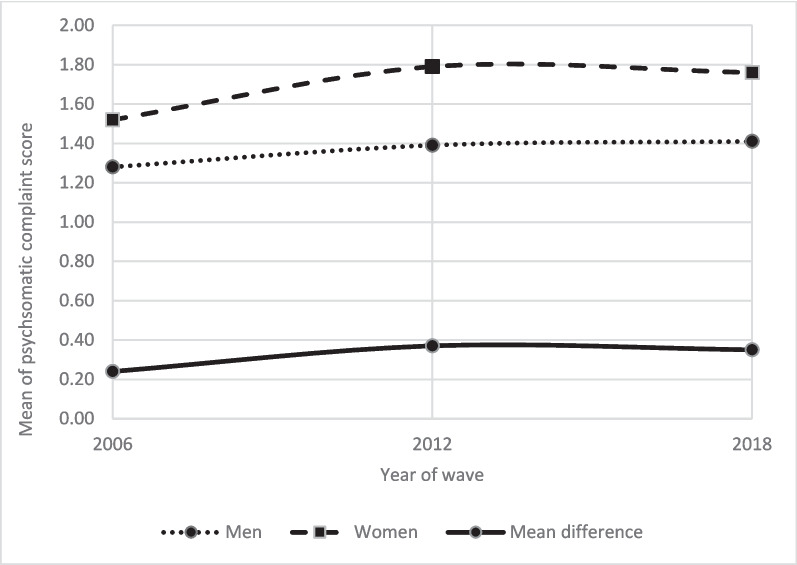
Time trend of means in psychosomatic complaints by gender from 2006 to 2018

As displayed in Table 2, of all participants in 2006, an overall numerical mean difference of Diff_male/female_ = 0.24 points and an effect size of 0.16 emerged between genders, with women having more symptoms, 95% CI [0.30; 0.41]. Regarding occupational subgroups, there were significant gender differences in white-collar high-skilled, white-collar low-skilled and blue-collar high-skilled jobs after controlling for the covariates age, parental status and working hours. The biggest difference was observed in white-collar high-skilled jobs with an effect size of 0.23 and 95% CI of [0.31; 0.46] when controlling for the mentioned covariates. Of all participants in 2012, an overall numerical mean difference of Diff_male/female_ = 0.37 points and an effect size of 0.23 (95% CI [0.42; 0.55]) emerged, with women having more symptoms. Regarding occupational subgroups, gender was a significant predictor as the exposure of primary interest in all occupational subgroups after controlling for the covariates age, parental status and working hours. The biggest difference is observed in white-collar high-skilled jobs with an effect size of 0.30 and 95% CI of [0.50; 0.66] when controlling for the mentioned covariates. Of all participants in 2018, an overall numerical mean difference of Diff_male/female_ = 0.35 points and an effect size of 0.22 emerged, with women having more symptoms (95% CI [0.37; 0.51]). Regarding occupational subgroups, gender was a significant predictor as the exposure of primary interest in white-collar high-skilled, white-collar low-skilled jobs and blue-collar low-skilled jobs after controlling for the covariates age, parental status and working hours. The repeated calculation of mean differences via t-tests stratified by collar and gender where trends were replicated can be found in the Appendix in Table A2. Overall, the effect size regarding gender differences rose from 2006 to 2012 and nearly stagnated from 2012 to 2018. The development of means and mean differences are displayed in Fig. 1.

**Table 2:** Mean differences of genders over years and collars

Wave 2006	Male Mean (SD)	Female Mean (SD)	Mean Difference	Adj.* P* Weighted Lin. Reg	95% CI	Effect size
Overall	1.28 (1.43)	1.52 (1.52)	0.24	** < .001**	[0.30; 0.41]	0.16
White-collar high-skilled	1.25 (1.44)	1.59 (1.53)	0.34	** < .001**	[0.31; 0.46]	0.23
White-collar low-skilled	1.29 (1.42)	1.41 (1.50)	0.12	** < .001**	[0.18; 0.45]	0.06
Blue-collar high-skilled	1.28 (1.39)	1.41 (1.51)	0.13	**.006**	[0.09; 0.55]	0.09
Blue-collar low-skilled	1.35 (1.46)	1.48 (1.54)	0.13	** < .001**	[0.23; 0.58]	0.09
**Wave 2012**						
Overall	1.39 (1.52)	1.76 (1.67)	0.37	** < .001**	[0.42; 0.55]	0.23
White-collar high-skilled	1.36 (1.52)	1.85 (1.69)	0.49	** < .001**	[0.50; 0.66]	0.30
White-collar low-skilled	1.51 (1.57)	1.60 (1.62)	0.09	**.029**	[0.02; 0.34]	0.08
Blue-collar high-skilled	1.34 (1.50)	1.68 (1.64)	0.34	** < .001**	[0.30; 0.77]	0.22
Blue-collar low-skilled	1.48 (1.53)	1.78 (1.64)	0.30	** < .001**	[0.30; 0.69]	0.19
**Wave 2018**						
Overall	1.41 (1.55)	1.76 (1.64)	0.35	** < .001**	[0.37; 0.51]	0.22
White-collar high-skilled	1.37 (1.54)	1.81 (1.65)	0.44	** < .001**	[0.40; 0.58]	0.28
White-collar low-skilled	1.62 (1.63)	1.68 (1.62)	0.06	**.006**	[0.08; 0.45]	0.04
Blue-collar high-skilled	1.39 (1.51)	1.52 (1.60)	0.13	.096	[−0.04; 0.48]	0.12
Blue-collar low-skilled	1.44 (1.54)	1.68 (1.62)	0.24	**.003**	[0.12; 0.59]	0.15

*P*- values and CIs refer to the direct effect of the outcome psychosomatic complaints after controlling for the covariates age (categorized in years), parental status (categorized in yes/no) and working hours (categorized in full-time/ part-time)

### Regression analysis and Interaction analyses of wave and gender

First, we estimated the model without interaction effects of gender and wave. As seen in Table 3, of all participants, women had a significantly higher symptom score with 0.42 points more on the psychosomatic complaint scale than men with a significant main effect for gender (95% CI [0.38; 0.46]). Wave had a significant main effect, meaning complaints significantly increased over the years. From 2006 to 2012, distress increased by 0.18 points (95% CI [0.14; 0.22]) and another 0.05 points to 2018 (95% CI [0.19; 0.27]). Working hours had a significant main effect, meaning people working full time had 0.34 more symptoms than participants working part-time (95% CI [0.29; 0.37]). Age showed a significant main effect (95% CI [−0.01; −0.003]); participants had 0.004 points less on their complaint scale with every year of life. Parental status showed no significant main effect (95% CI [−0.05; 0.03]).

**Table 3:** Weighted regression analysis

Variable	Coefficient	T	P	95% CI
Gender				
Male	Ref. = 1	-	-	-
Female	.42	21.20	< .001	[0.38; 0.46]
Working hours				
Fulltime (min. 36 h)	Ref. = 1	-	-	-
Not fulltime	-.34	16.37	< .001	[0.29/0.37]
Age	-.004	−5.63	< .001	[−0.01/−0.003]
Parental status	Ref. = 1	-	-	-
Having no children	-.01	−0.50	.621	[−0.05/0.03]
Collar				
White-collar high-skilled	Ref. = 1	-	-	-
White-collar low-skilled	-.03	−1.49	.137	[0.08/0.01]
Blue-collar high-skilled	-.08	−3.25	.001	[−0.13/−0.03]
Blue-collar low-skilled	.04	1.26	.208	[−0.02/0.09]
Wave				
2006	Ref. = 1	-	-	-
2012	.18	9.14	< .001	[0.14/0.22]
2018	.23	10.64	< .001	[0.18/0.27]

Then, we used interaction analysis to test if the gender differences significantly changed over time. Results of the interactions are displayed in Table 4. Regarding the interaction effect, significant interaction effects emerged for each year. The effect of gender on score in year 2006 was used as a reference. In 2012, the gender difference increases by 0.12 points (*P* = 0.002) in comparison to 2006. In 2018, the increase is 0.11 (*P* = 0.005). As this number is lower than in 2012, there was a slight decrease in size of difference compared in 2018. However, as confidence intervals of 2012 and 2018 are overlapping, there is a significant increase from 2006 to 2012 but a stagnation (or no significant difference in size) from 2012 to 2018.

**Table 4:** Results of interactions in weighted interaction analysis gender x wave on psychosomatic complaints including covariates working hours and age

Variable	Coefficient	T	P	95% CI
Interaction Gender x Wave				
Female × 2006	Ref. = 1	-	-	-
Female × 2012	.12	3.14	.002	0.05/0.20
Female × 2018	.11	2.73	.006	0.03/0.20

The same covariates were included as in the weighted regression analysis but are not shown. Further results can be found in the Appendix in table A3

## Discussion

Using three waves (2006, 2012, and 2018) of the nationwide German employment survey, we analysed gender differences in psychosomatic complaints across occupational categories. We classified occupations into four subgroups: high-skilled and low-skilled positions in both white-collar and blue-collar sectors. Our analysis revealed a persistent and partly widening gender disparity in psychosomatic complaints across occupational subgroups. Women reported significantly higher levels of complaints compared to men, independent of age, parental status, and working hours but especially pronounced in high-skilled white-collar occupations. This gender gap expanded over the twelve-year period, confirming and extending previous research on gender differences in workplace-related psychosomatic symptoms [6–10].

Regarding the overall trend over time, the magnitude of gender differences in psychosomatic complaints significantly increased from 2006 to 2012, with effect sizes rising from 0.16 to 0.23, before plateauing by 2018. Additionally, weighted regression models controlling for age and full-time/part-time employment status in interaction analyses revealed a significant interaction effect in psychosomatic complaints by gender and survey wave. Specifically, the gender gap significantly expanded between 2006 and 2012 but stabilized from 2012 to 2018.

The use of weighted regression in interaction analyses introduced some variation compared to manually calculated effect sizes; however, the overall trend remained consistent. The increase in gender differences from 2006 to 2012 supports the hypothesis of widening disparities over time, driven in part by the growing proportion of white-collar high-skilled workers, who exhibited the most pronounced gender differences in psychosomatic complaints. In contrast, the subsequently observed stagnation from 2012 to 2018 challenges this hypothesis. These findings indicate that the gender gap in psychosomatic complaints has either stabilized or further widened, with the largest disparities consistently observed among high-skilled white-collar workers.

The gender gap in psychosomatic complaints was evident across all occupational subgroups, with varying degrees of severity. Among white-collar high-skilled workers, the numerical disparities in mean psychosomatic complaint scores were consistently the largest across survey years, with effect sizes ranging from 0.22 to 0.30, classified as small according to Cohen's criteria [42]. In contrast, other subgroups, such as white-collar low-skilled, blue-collar high-skilled, and blue-collar low-skilled workers, showed fluctuating effect sizes over the years, ranging from marginal to small. This analysis indicates that white-collar high-skilled occupations consistently present the highest risk for gender disparities in psychosomatic complaints, a trend observed from 2006 to 2018.

### Explanations for occupational gender differences and trends

Several factors may explain these findings. First, while the gender pay gap has narrowed in recent decades, this reduction has been less pronounced in high-skilled and high-income occupations [20, 24]. Both perceived and actual unfair treatment or inadequate compensation can contribute to physical and psychological health issues, including burnout symptoms similar to those examined in this study. Income, as emphasized in Siegrist's effort-reward imbalance (ERI) model [43], represents a crucial reward for employee effort. This model has demonstrated consistent associations with psychosomatic symptoms across various occupations [44, 45]. Future studies should explore the relationship between ERI and gender-specific psychosomatic symptoms across occupational categories.

A complementary explanation emerges from Herlitz et al.'s meta-analysis [46], which revealed more pronounced gender differences in psychological measures within countries characterized by higher living standards—strong economies, advanced education, and greater gender equality—a phenomenon termed the "gender-equality paradox" [47]. In these contexts, women reported elevated depressive symptoms compared to men, while men exhibited higher overall anger levels. Women also reported greater happiness, though this difference diminished in countries with superior living conditions. While these studies did not differentiate occupational subgroups, their findings align with our results. The enhanced gender differences in psychosomatic complaints among high-skilled white-collar workers, who typically enjoy better living conditions through higher income and education, mirror this broader pattern. The resource theory proposed by Foa and Foa [48] suggests that economic pressure reduces gender differences in job preferences, while resource-rich environments enable pursuit of occupations aligned with personality traits or preferences, potentially including more psychosocially demanding roles. External factors, such as avoiding male-dominated professions due to concerns about sexist behavior, may also influence occupational choices and subsequent psychosomatic complaints among women. Further investigation is needed to understand occupational gender preferences in societies with higher living standards.

A third factor involves women's disproportionate caregiving responsibilities [25], rooted in societal expectations and traditions, which create additional psychosomatic health stressors beyond occupational demands. While this imbalance likely affects all occupational subgroups, it may not fully account for occupation-specific gender differences in psychosomatic health. White-collar high-skilled positions may offer less supportive conditions for managing caregiving responsibilities, potentially contributing to observed health outcome disparities, despite greater financial resources for household assistance. However, as long as caregiving organization and management remain predominantly women's responsibility within partnerships, associated stress may persist. Further research should examine how caregiving responsibilities interact with occupational factors to influence gender differences in psychosomatic health.

### Further findings: Increase in psychosomatic complaints over time and benefits of part-time work

Not only did the difference in psychosomatic complaints between genders increase between 2006 and 2012, but the number of psychosomatic complaints themselves also rose. This trend aligns with findings indicating an increase in depressive symptoms, particularly somatic symptoms, among younger birth cohorts, both in Germany [49] and e.g. the United States of America [50]. Several factors may contribute to this phenomenon, including an increase in certain chronic illnesses and morbidity among younger generations [51, 52], which often co-occur with depressive symptoms [53, 54], as well as an increase in health knowledge and accessibility to mental health care.

Additionally, working part-time (less than 36 h/week) was associated with decreased psychosomatic complaints. This observation is consistent with findings suggesting that long working hours (more than 35 h/week) are associated with diminished well-being, although the literature is inconsistent [37, 38]. Individuals may have greater opportunities to fulfill multiple roles (e.g., parent, friend, soccer player), not solely the role of a worker, which has been shown to be protective for mental health, as outlined in the role-enhancement hypothesis [39, 55]. If women’s work patterns shift toward full-time employment similar to men's, gender differences in psychosomatic symptoms might be increased. However, despite the increasing participation of women in the workforce [21], the proportion of women in Germany working full-time remained nearly stable at around 34% between 2002 and 2022 [22, 23]. Comparison with our sample data revealed a similar stagnation over the years, albeit with a higher proportion of women working full-time. Men, in turn, stagnated in their share of working part-time as well (22, 23, Table A1 in the Appendix).

### Relevance for the population

Our analyses incorporated controls for age, parental status, and working hours. Comparative analyses without these controls revealed similar but attenuated trends, suggesting that the observed differences extend beyond these demographic factors, and their exclusion would underestimate the true differences.

While the effect sizes of mean differences were modest, small population-level effects can produce substantial impacts at distribution extremes [56, 57]. The critical differences emerge in the distribution area of high psychosomatic complaint frequencies. Exceeding certain symptom thresholds may trigger work absence or meet criteria for depressive episodes, which share symptom patterns with our study measures [58]. In large populations, such as the German workforce, slight increases in mean psychosomatic burden can significantly affect the number of individuals crossing critical thresholds. For instance, a mean difference of 0.40 could translate to thousands of additional individuals experiencing severe symptom burden within a population of millions. Though individual symptom tolerance and coping thresholds vary, these distributional differences may explain gender disparities in work absenteeism and healthcare-seeking behaviours.

### Limitations

There are several limitations that need to be considered. In 2006, participants were asked “Tell me, if the following health complaints appeared at or immediately after work”; in 2012 and 2018, participants were asked “Tell me, if the following health complaints appeared at work or on workdays. We are interested in complaints that appeared frequently”. A slight change of the choice of words may influence the answering of a question [59]. Possibly, in 2006, participants thought about a different time period when they experience the symptoms due to change of wording. However, since we were interested in the relative differences between genders over time and not in the absolute numeric values or absolute changes in values over time, we considered the data to be comparable for our research questions. Even if the questioning changed the answers, this effect should appear with both genders, so the time trend in gender differences could not be explained by it. Additionally, as questions are consistent in sequence over the survey waves and all have an open question style, other priming effects are unlikely to be present. Further research with different data sets and more matching questions over the years should be used to validate the findings.

The psychosomatic complaints score and the selection of items used were developed by the authors and are not based on a validated complaints scale. Using a validated scale would have been more appropriate to ensure the validity and reliability of the results and is recommended for future research.

The reliability and validity of self-reporting may vary across symptoms. For instance, pain such as headaches may be easier to self-assess compared to irritability, which requires a higher level of introspection and perceptual acuity. Additionally, the survey does not differentiate between trivial and severe symptoms. In terms of generalizability, being a survey-based study, this sample may be susceptible to selectivity and non-response bias, as individuals who are healthier are more inclined to participate in interviews [60, 61].

Although parental status was taken into account, the number and age of children were not included in the analysis due to insufficient responses regarding this information. In 2006, only a small number of participants provided answers to the questions about the number and age of their children. However, caring for more than one child, particularly young children, may increase the demand for care work and related psychosomatic stress, especially for women. This important distinction was not addressed in the study. This may contribute to the unexpected result of parenthood not showing an important impact on distress in the analysis, which was found elsewhere in research [62]. Further research considering age and number of children should take these effects into account.

Another key limitation of the current study is its reliance on only three time points. This restricts our ability to determine whether gender disparities will continue to widen or stabilize, making trend predictions largely speculative. Future research incorporating additional time points would enable more precise analysis of these temporal dynamics.

### Opportunities for further research

Using more recent data, it is imperative to investigate whether the gender difference in psychosomatic complaints remains stable or potentially exacerbates due to evolving dynamics in modern workplaces. With the increasing prevalence of higher education [63–66], greater participation of females [67, 68], and the growth of white-collar high-skilled occupations [65] observed in many countries, international comparisons should be conducted to analyse trends across different countries. If similar trends appear across countries, this might suggest common underlying causes; if not, it raises questions about differences in work environments and gender policies. Furthermore, it would be intriguing to assess whether high-income countries exhibit greater disparities in psychosomatic complaints at work, as suggested by previous findings on gender differences in overall health and emotional well-being across countries [46].

## Conclusion

Gender differences among employed Germans have been consistently observed across all occupational subgroups from 2006 to 2018. Notably, women employed in white-collar high-skilled positions exhibit a heightened risk of experiencing more symptoms compared to their male counterparts. Furthermore, gender differences within the population increased from 2006 to 2012, followed by a period of stagnation from 2012 to 2018. Further investigation is warranted to elucidate the underlying reasons for these disparities and trends, as well as to conduct cross-national comparisons with other countries, thereby providing valuable insights into the broader context of gender differences in psychosomatic health at work.

## Supplementary Information

Additional file 1.

Additional file 2.

## Data Availability

The data that support the findings of this study are available from Data Research Centre at the Federal Institute for Vocational Training and Education (BIBB-FDZ), but restrictions apply to the availability of these data, which were used under license for the current study, and so are not publicly available. Data are however freely available for academic purposes in a public repository on request after having signed an agreement with the owner (https://www.bibb.de/de/1403.php). SUFs are distributed directly via the BIBB-FDZ. For this purpose, an application form (available online via https://www.bibb.de/dokumente/pdf/BIBB_FDZ_Antrag_SUF_FDZ_deutsch.pdf) must be completed, signed and sent to the BIBB-FDZ by post (BIBB - Bundesinstitut für Berufsbildung Arbeitsbereich 1.5: Forschungsdatenzentrum, Postfach 20 12 64, 53142 Bonn, Germany) or e-mail (fdz@bibb.de).
