# Supplementary material for: Gender differences in psychosomatic complaints across occupations and time from 2006 to 2018 in Germany: a repeated cross-sectional study
Source: BMC Public Health. 2025 Feb 1;25:409. doi: 10.1186/s12889-025-21462-8 (PMC11786428; doi:10.1186/s12889-025-21462-8)
Supplement: Supplementary file 1 — Additional file 1. [file 12889_2025_21462_MOESM1_ESM.docx]

Appendix

Part A: Results

Figure AF1. *Scree plot of eigenvalues after factor.*


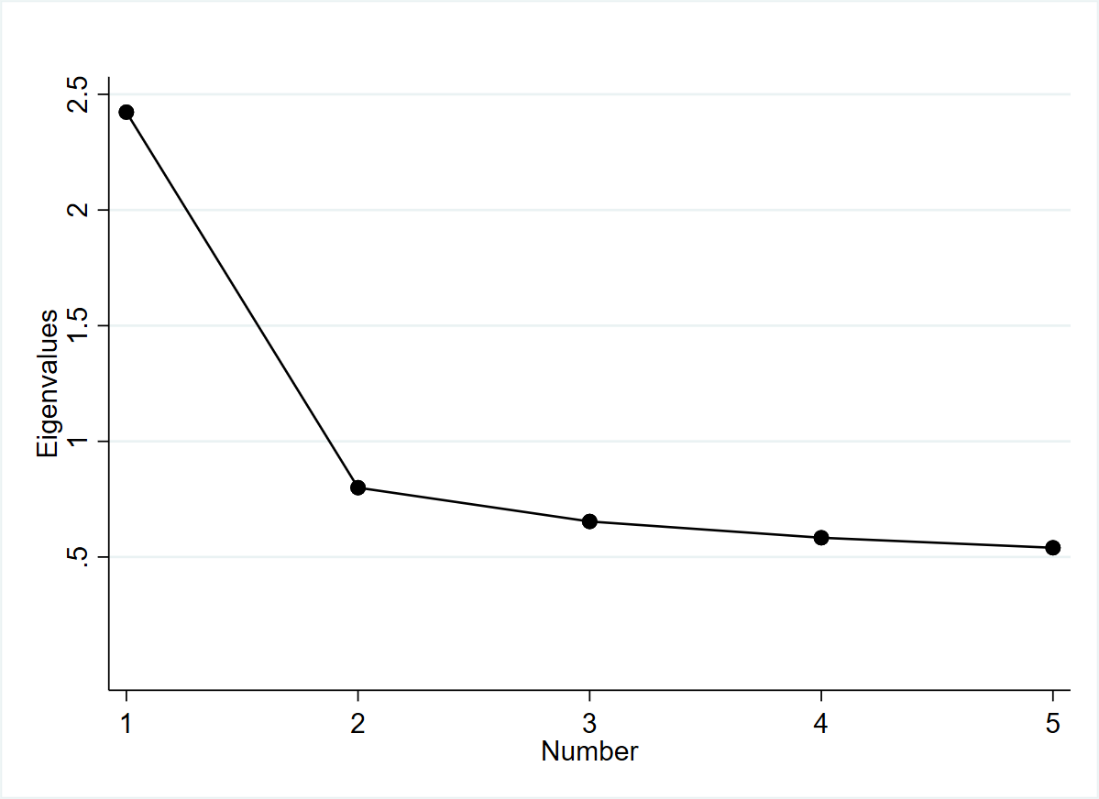


Figure AF2. *Plot showing the results of the parallel analysis of a PCA on simulated*

*data with one true component underlying 5 variables.*


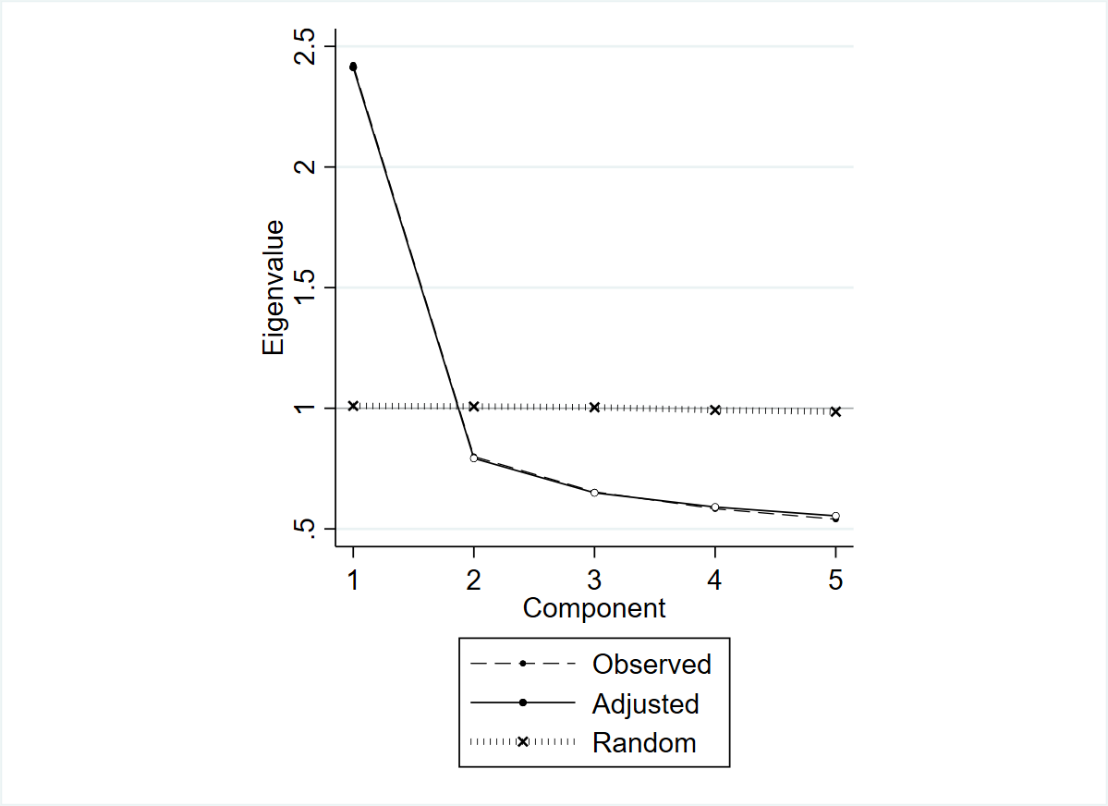


Table A1. *Descriptive data of mean age, collar proportion and women working fulltime over the years.*

|  | 2006 | 2012 | 2018 |
| --- | --- | --- | --- |
| Age in years (Mean/ SD) | 41.31 / 10.46 | 46.06 / 10.70 | 47.22 / 11.31 |
| White-collar high-skilled (N/ %) | 10533 / 53.13 % | 10786 / 55.07 % | 12516 / 63.97 % |
| White-collar low-skilled (N/ %) | 4199 / 21.18 % | 4185 / 21.37 % | 3336 / 17.05 % |
| Blue-collar high-skilled (N/ %) | 2828 / 14.27 % | 2496 / 12.74 % | 1913 / 9.78 % |
| Blue-collar low-skilled (N/ %) | 2264 / 11.42 % | 2119 / 10.82 % | 1801 / 9.20 % |
| Proportion of women working fulltime (%) | 47.47 % | 53.08 % | 50.35 % |
| Proportion of men working fulltime (%) | 88.36 % | 88.97 % | 86.37 % |

| **Wave / Year** | **2006** | **2012** | **2018** |
| --- | --- | --- | --- |
| Overall | .000 | .000 | .000 |
| White-collar high-skilled | .000 | .000 | .000 |
| White-collar low-skilled | .018 | .113 | .315 |
| Blue-collar high-skilled | .109 | .000 | .223 |
| Blue-collar low-skilled | .052 | .000 | .007 |

Table A2. *P-values of mean differences in psychosomatic complaint score after t-test/ Bootstrap 2000rep of means between genders by collar.*

Table A3. *Interaction analysis further results.*

| **Variable** | **Coefficient** | **T** | **P** | **95 % CI** |
| --- | --- | --- | --- | --- |
| Gender |  |  |  |  |
| Male | Ref. = 1 | - | - | - |
| Female | .35 | 12.90 | <.001 | [0.30/0.40] |
| Working hours |  |  |  |  |
| Fulltime (min. 36hours) | Ref. = 1 | - | - | - |
| Not fulltime | -.33 | 16.41 | <.001 | [0.29/0.37] |
| Age | -.004 | -5.37 | <.001 | [-0.01/-0.003] |
| Parental status | Ref. = 1 | - | - | - |
| Having no children | -.01 | -0.44 | .660 | [-0.04/0.03] |
| Collar |  |  |  |  |
| White-collar high-skilled | Ref. = 1 | - | - | - |
| White-collar low-skilled | -.03 | -1.45 | .146 | ][0.08/0.01] |
| Blue-collar high-skilled | -.08 | -3.22 | .001 | [-0.01/-0.03] |
| Blue-collar low-skilled | .04 | 1.48 | .140 | [-0.01/0.10] |
| Wave |  |  |  |  |
| 2006 | Ref.= 1 | - | - | - |
| 2012 | .13 | 4.69 | <.001 | [0.07/0.18] |
| 2018 | .18 | 6.23 | <.001 | [0.12/0.24] |
| Interaction Gender x Wave |  |  |  |  |
| Female x 2006 | Ref.= 1 | - | - | - |
| Female x 2012 | .12 | 3.14 | .002 | [0.05/0.20] |
| Female x 2018 | .11 | 2.73 | .006 | [0.03/0.20 |

Table A4. *Sensitivity analysis of mean differences of genders by wave and collar using additional physical variables from the original questionnaire*

| **Variable** | **Coefficient** | **T** | **P** | **95 % CI** |
| --- | --- | --- | --- | --- |
| Gender |  |  |  |  |
| Male | Ref. = 1 | - | - | - |
| Female | .64 | 13.74 | <.001 | 0.55/0.74 |
| Working hours |  |  |  |  |
| Fulltime (min. 36 hours) | Ref. = 1 | - | - | - |
| Not fulltime | .57 | 16.07 | <.001 | 0.50/0.64 |
| Age | .01 | 6.34 | <0.001 | 0.01/0.01 |
| Parental status | Ref. = 1 | - | - | - |
| Having no children | -.06 | -1.88 | .06 | -0.12/0.003 |
| Wave |  |  |  |  |
| 2006 | Ref.= 1 | - | - | - |
| 2012 | .22 | 4.48 | <.001 | 0.12/0.31 |
| 2018 | .34 | 6.58 | <.001 | 0.24/0.44 |
| Interaction Gender x Wave |  |  |  |  |
| Female x 2006 | Ref.= 1 | - | - | - |
| Female x 2012 | .25 | 3.63 | <.001 | 0.11/0.38 |
| Female x 2018 | .20 | 2.68 | .007 | 0.05/0.34 |

Table A5. *Sensitivity analysis of interaction analysis using additional physical variables from the original questionnaire*

| **Wave 2006** | **Male Mean (SD)** | **Female Mean (SD)** | **Mean Difference** | **Adj. *P***  **Weighted Lin. Reg.** | **Effect size** |
| --- | --- | --- | --- | --- | --- |
| Overall | 2.51 (2.50) | 3.01 (2.55) | 0.5 | **<.001** | 0.20 |
| White-collar high-skilled | 2.25 (2.34) | 3.01 (2.51) | 0.76 | **<.001** | 0.31 |
| White-collar low-skilled | 2.40 (2.40) | 2.90 (2.56) | 0.50 | **<.001** | 0.20 |
| Blue-collar high-skilled | 2.87 (2.55) | 3.20 (2.65) | 0.33 | **<.001** | 0.12 |
| Blue-collar low-skilled | 2.90 (2.63) | 3.30 (2.71) | 0.40 | **<.001** | 0.15 |
| **Wave 2012** |  |  |  |  |  |
| Overall | 2.81 (2.67) | 3.58 (2.90) | 0.77 | **<.001** | 0.28 |
| White-collar high-skilled | 2.56 (2.59) | 3.63 (2.89) | 1.07 | **<.001** | 0.39 |
| White-collar low-skilled | 2.91 (2.71) | 3.37 (2.84) | 0.46 | **<.001** | 0.17 |
| Blue-collar high-skilled | 3.06 (2.69) | 3.67 (3.01) | 0.61 | **<.001** | 0.21 |
| Blue-collar low-skilled | 3.23 (2.81) | 3.92 (2.99) | 0.69 | **<.001** | 0.24 |
| **Wave 2018** |  |  |  |  |  |
| Overall | 2.81 (2.70) | 3.55 (2.85) | 0.74 | **<.001** | 0.27 |
| White-collar high-skilled | 2.58 (2.58) | 3.55 (2.84) | 0.97 | **<.001** | 0.36 |
| White-collar low-skilled | 3.14 (2.88) | 3.51 (2.87) | 0.37 | **<.001** | 0.13 |
| Blue-collar high-skilled | 3.23 (2.80) | 3.51 (2.80) | 0.23 | .154 | 0.08 |
| Blue-collar low-skilled | 3.16 (2.83) | 3.71 (3.00) | 0.55 | **.001** | 0.19 |

*Note: Analysis used were the same as in the analysis for the research question: weighted linear regression analysis and Cohen’s d.*
