# Supplementary material for: Gender differences in psychosomatic complaints across occupations and time from 2006 to 2018 in Germany: a repeated cross-sectional study
Source: BMC Public Health. 2025 Feb 1;25:409. doi: 10.1186/s12889-025-21462-8 (PMC11786428; doi:10.1186/s12889-025-21462-8)
Supplement: Supplementary file 2 — Additional file 2. [file 12889_2025_21462_MOESM2_ESM.docx]

# Appendix Part B

# Do-File for Reproduction using STATA MP

## 1. Step: Preparation of the dataset of 2006

## *Dofile Aufbau 2006 Deutschland

## use "C:\Users\julia\Desktop\MHH WiMi\Daten und Fragebögen\ZA4820 - 2006\ZA4820_v3-0-0.dta"

## *collar erstellen. Achtung: Hier 4Steller; 2012 und 2018 war 3Steller

## gen collar = 99

## *White collar high skilled Gruppe erstellen

## replace collar = 1 if (f100_i88 >= 1110) & (f100_i88 <=3999)

## *White collar low skilled Gruppe erstellen

## replace collar = 2 if (f100_i88 >= 4000) & (f100_i88 <=5999)

## *Blue collar high skilled Gruppe erstellen

## replace collar = 3 if (f100_i88 >= 6000) & (f100_i88 <=7999)

## *Blue collar low skilled Gruppe erstellen

## replace collar = 4 if (f100_i88 >= 8000) & (f100_i88 <=9999)

## drop if collar ==99

## *91 gelöscht

## *Label für collar Varianten in Variable eintragen

## *Missings bleiben 99

## *4-Schritt-collar

## label define collar 1 "high skilled white collar" 2 "low skilled white collar" 3 "high skilled blue collar" 4 "low skilled blue collar" 5 "Streitkräfte"

## tab collar

## tab collar, nolabel

## *2-Schritt-collar

## gen collar2 = 99

## replace collar2 = 1 if (collar >= 1)

## replace collar2 = 2 if (collar >= 3)

## label define collar2 1 "white" 2 "blue"

## *Dummy für Gender erstellen

## gen GenderDummy = 0

## replace GenderDummy = 1 if s1 == 2

## *Dummy für Collar erstellen

## gen CollarDummy = collar2

## replace CollarDummy = 0 if collar2 == 1

## replace CollarDummy = 1 if collar2 == 2

## *Dummy für Kindstatus erstellen

## gen Kindstatus = f1604

## gen KindstatusDummy = Kindstatus

## replace KindstatusDummy = 0 if Kindstatus == 1

## replace KindstatusDummy = 1 if Kindstatus == 2

## drop if Kindstatus == 9

## *Belastungen netter labeln

## gen Kopfschmerzen = f1500_07

## gen Schlafstörungen = f1500_14

## gen Müdigkeit = f1500_15

## gen Reizbarkeit = f1500_18

## gen Niedergeschlagenheit = f1500_19

## *Achtung: Körperl. und emot. Erschöpfung 2006 fehlend

## *Optional:

## gen RückenKreuzschmerzen = f1500_01

## gen SchulterNackenschmerzen = f1500_02

## gen Hüftschmerzen = f1500_04

## gen HerzschmerzenEngeStiche = f1500_08

## gen HautreizungenJuckreiz = f1500_13

## gen MagenVerdauungsbeschwerden = f1500_16

## gen HörverschlechterungOhrgeräusche = f1500_17

## gen Schwindel = f1500_20

## *KopfschmerzenDummy erstellen für logsitische Regression später

## gen KopfschmerzenDummy = Kopfschmerzen

## replace KopfschmerzenDummy = 1 if Kopfschmerzen == 1

## replace KopfschmerzenDummy = 0 if Kopfschmerzen == 2

## *SchlafstörungenDummy erstellen für logsitische Regression später

## gen SchlafstörungenDummy = Schlafstörungen

## replace SchlafstörungenDummy = 1 if Schlafstörungen == 1

## replace SchlafstörungenDummy = 0 if Schlafstörungen == 2

## *MüdigkeitDummy erstellen für logsitische Regression später

## gen MüdigkeitDummy = Schlafstörungen

## replace MüdigkeitDummy = 1 if Müdigkeit == 1

## replace MüdigkeitDummy = 0 if Müdigkeit == 2

## *ReizbarkeitDummy erstellen für logsitische Regression später

## gen ReizbarkeitDummy = Reizbarkeit

## replace ReizbarkeitDummy = 1 if Reizbarkeit == 1

## replace ReizbarkeitDummy = 0 if Reizbarkeit == 2

## *NiedergeschlagenheitDummy erstellen für logsitische Regression später

## gen NiedergeschlagenheitDummy = Niedergeschlagenheit

## replace NiedergeschlagenheitDummy = 1 if Niedergeschlagenheit == 1

## replace NiedergeschlagenheitDummy = 0 if Niedergeschlagenheit == 2

## *Arbeitszeitgruppen erstellen

## gen Arbeitszeitgruppe = 999

## replace Arbeitszeitgruppe = 1 if (az <= 10)

## replace Arbeitszeitgruppe = 2 if (az >10) & (az <=20)

## replace Arbeitszeitgruppe = 3 if (az >20) & (az <=30)

## replace Arbeitszeitgruppe = 4 if (az >30) & (az <=120)

## *Belastungsscore erstellen aus 0-1-Belastungsvariablen

## gen Kopfschmerzen01 = 0

## gen Schlafstörungen01 = 0

## gen Müdigkeit01 = 0

## gen Reizbarkeit01 = 0

## gen Niedergeschlagenheit01 = 0

## replace Kopfschmerzen01 = 1 if (f1500_07 <2)

## replace Schlafstörungen01 = 1 if (f1500_14 <2)

## replace Müdigkeit01 = 1 if (f1500_15 <2)

## replace Reizbarkeit01 = 1 if (f1500_18 <2)

## replace Niedergeschlagenheit01 = 1 if (f1500_19 <2)

## gen BelastungsscoreKurz = Kopfschmerzen01+ Schlafstörungen01+ Müdigkeit01+ Reizbarkeit01+ Niedergeschlagenheit01

## gen wave = 2006

## gen wave2006 = 1

## *Variablen angleichen für das Mergen

## gen S1 = s1

## gen Intnr = idnum

## gen F200 = f200

## gen AZ = az

## gen F1604 = f1604

## gen F518 = f518

## gen F16000f1600

## *Eingetragene Partnerschaft zu Ehe machen

## replace F1600 = 1 if = (f1600 <5)

## gen Familienstatus = f1600

## *Missings

## *Geschlecht

## tab S1

## *keine Missings bei Geschlecht, keine Löschungen

## *Belastungen

## tab f1500_07

## tab f1500_07, nolabel

## drop if f1500_07 == 9

## *8 gelöscht

## tab f1500_14

## drop if f1500_14 == 9

## *9 gelöscht

## tab f1500_15

## drop if f1500_15 == 9

## *11 gelöscht

## drop if f1500_15 == 9

## *0 gelöscht

## drop if f1500_18 == 9

## *6 gelöscht

## *Optional

## *Arbeitszeit:

## tab az

## *keine Missings in Arbeitzeit, keine Löschungen

## *Alter

## tab zpalter

## drop if zpalter == 0

## *18 gelöscht

## save "C:\Users\julia\Desktop\MHH WiMi\Daten 2006 nach Aufbau(neu).dta"

## 2. Step: Preparation of the dataset of 2012

use "C:\Users\julia\Desktop\MHH WiMi\Daten und Fragebögen\ZA5657 - 2012\ZA5657_v6-0-0.dta"

*collar Variable erstellen

gen collar = 99

*Jobs in Gruppen einteilen innerhalb von collar

*High skilled white collar hinein codieren

replace collar = 1 if (F100_isco88_3d >= 100) & (F100_isco88_3d <=399)

*Low skilled white collar reincodieren

replace collar = 2 if (F100_isco88_3d >= 400) & (F100_isco88_3d <=599)

*High skilled blue collar reincodieren

replace collar = 3 if (F100_isco88_3d >= 600) & (F100_isco88_3d <=799)

*Low skilled blue collar reincodieren

replace collar = 4 if (F100_isco88_3d >= 800) & (F100_isco88_3d <=999)

*Streitkräfte in diesem Datensatz nicht vorhanden, daher kein extra Umgang

*Unklare Berufsbezeichnung ausschließen

drop if collar == 99

*-->113 gelöscht

*Label für collar Varianten in Variable eintragen

*Missings bleiben 99

*4-Schritt-collar

label define collar 1 "high skilled white collar" 2 "low skilled white collar" 3 "high skilled blue collar" 4 "low skilled blue collar" 5 "Streitkräfte"

tab collar

tab collar, nolabel

*2-Schritt-collar

gen collar2 = 99

replace collar2 = 1 if (collar >= 1)

replace collar2 = 2 if (collar >= 3)

label define collar2 1 "white" 2 "blue"

*Dummy für Gender erstellen

gen GenderDummy = 0

replace GenderDummy = 1 if S1 == 2

*Dummy für Collar erstellen

gen CollarDummy = collar2

replace CollarDummy = 0 if collar2 == 1

replace CollarDummy = 1 if collar2 == 2

*Dummy für Kindstatus erstellen

gen Kindstatus = F1604

gen KindstatusDummy = Kindstatus

replace KindstatusDummy = 0 if Kindstatus == 1

replace KindstatusDummy = 1 if Kindstatus == 2
drop if Kindstatus == 9

*Belastungen llabeln

gen Kopfschmerzen = F1500_07

gen Schlafstörungen = F1500_14

gen Müdigkeit = F1500_15

gen Reizbarkeit = F1500_18

gen Niedergeschlagenheit = F1500_19

gen KörperlicheErschöpfung = F1500_21

gen EmotionaleErschöpfung = F1500_22

gen RückenKreuzschmerzen = F1500_01

gen SchulterNackenschmerzen = F1500_02

gen Hüftschmerzen = F1500_04

gen HerzschmerzenEngeStiche = F1500_08

gen HautreizungenJuckreiz = F1500_13

gen MagenVerdauungsbeschwerden = F1500_16

gen HörverschlechterungOhrgeräusche = F1500_17

gen Schwindel = F1500_20

*Vollzeit/Teilzeit Variable erstellen

gen Vollzeit35=99

replace Vollzeit35 = 1 if az>35

replace Vollzeit35 = 0 if az<36

tab Vollzeit35

label define Vollzeit35 0 "nein" 1 "ja"

*KopfschmerzenDummy erstellen für logsitische Regression später

gen KopfschmerzenDummy = Kopfschmerzen

replace KopfschmerzenDummy = 1 if Kopfschmerzen == 1

replace KopfschmerzenDummy = 0 if Kopfschmerzen == 2

*SchlafstörungenDummy erstellen für logsitische Regression später

gen SchlafstörungenDummy = Schlafstörungen

replace SchlafstörungenDummy = 1 if Schlafstörungen == 1

replace SchlafstörungenDummy = 0 if Schlafstörungen == 2

*MüdigkeitDummy erstellen für logsitische Regression später

gen MüdigkeitDummy = Schlafstörungen

replace MüdigkeitDummy = 1 if Müdigkeit == 1

replace MüdigkeitDummy = 0 if Müdigkeit == 2

*ReizbarkeitDummy erstellen für logsitische Regression später

gen ReizbarkeitDummy = Reizbarkeit

replace ReizbarkeitDummy = 1 if Reizbarkeit == 1

replace ReizbarkeitDummy = 0 if Reizbarkeit == 2

*NiedergeschlagenheitDummy erstellen für logsitische Regression später

gen NiedergeschlagenheitDummy = Niedergeschlagenheit

replace NiedergeschlagenheitDummy = 1 if Niedergeschlagenheit == 1

replace NiedergeschlagenheitDummy = 0 if Niedergeschlagenheit == 2

*KörperlicheErschöpfungDummy erstellen für logsitische Regression später

gen KörperlicheErschöpfungDummy = KörperlicheErschöpfung

replace KörperlicheErschöpfungDummy = 1 if KörperlicheErschöpfung == 1

replace KörperlicheErschöpfungDummy = 0 if KörperlicheErschöpfung == 2

*EmotionaleErschöpfungDummy erstellen für logsitische Regression später

gen EmotionaleErschöpfungDummy = EmotionaleErschöpfung

replace EmotionaleErschöpfungDummy = 1 if EmotionaleErschöpfung == 1

replace EmotionaleErschöpfungDummy = 0 if EmotionaleErschöpfung == 2

*Belastungsscore erstellen aus 0-1-Belastungsvariablen

gen Kopfschmerzen01 = 0

gen Schlafstörungen01 = 0

gen Müdigkeit01 = 0

gen Reizbarkeit01 = 0

gen Niedergeschlagenheit01 = 0

gen KörperlicheErschöpfung01 = 0

gen EmotionaleErschöpfung01 = 0

replace Kopfschmerzen01 = 1 if (F1500_07 <2)

replace Schlafstörungen01 = 1 if (F1500_14 <2)

replace Müdigkeit01 = 1 if (F1500_15 <2)

replace Reizbarkeit01 = 1 if (F1500_18 <2)

replace Niedergeschlagenheit01 = 1 if (F1500_19 <2)

replace KörperlicheErschöpfung01 = 1 if (F1500_21 <2)

replace EmotionaleErschöpfung01 = 1 if (F1500_22 <2)

gen Belastungsscore = Kopfschmerzen01+ Schlafstörungen01+ Müdigkeit01+ Reizbarkeit01+ Niedergeschlagenheit01+ KörperlicheErschöpfung01+ EmotionaleErschöpfung01

*bis hier wie 2018*

gen BelastungsscoreKurz = Kopfschmerzen01+ Schlafstörungen01+ Müdigkeit01+ Reizbarkeit01+ Niedergeschlagenheit01

gen wave = 2012

gen wave2012 = 1

*Variablen ergänzen fürs mergen

gen zpalter = Zpalter

*Missings

*Geschlecht

tab S1

*keine Missings bei Geschlecht, keine Löschungen

*Collar nicht zuordbar wurde oben schon gelöscht: 113 Fälle gelöscht

*Belastungen

tab F1500_07

tab F1500_07, nolabel

drop if F1500_07 == 9

*39 gelöscht

tab F1500_14

drop if F1500_14 == 9

*21 gelöscht

tab F1500_15

drop if F1500_15 == 9

*6 gelöscht

tab F1500_18

drop if F1500_18 == 9

*13 gelöscht

tab F1500_19

drop if F1500_19 == 9

*6 gelöscht

tab F1500_21

drop if F1500_21 == 9

*9 gelöscht

tab F1500_22

drop if F1500_22 == 9

*16 gelöscht

*110 wegen Belastungsmissings gelöscht

*Alter:

tab Zpalter

drop if Zpalter == 9999

*--> 61 wegen fehlender Altersdaten gelöscht

*Arbeitszeit:

tab az

drop if az>120

*--> 48 wegen fehlender Arbeitszeit gelöscht

save "C:\Users\julia\Desktop\MHH WiMi\Daten 2012 nach Aufbau(neu).dta"

## 3. Step: Prepartion of the dataset of 2018:

************

***AUFBAU***

************

use "C:\Users\julia\Desktop\MHH WiMi\Daten und Fragebögen\ZA7574 - 2018\ZA7574_v1-0-0"

*collar Variable erstellen

gen collar = 99

*Jobs in Gruppen einteilen innerhalb von collar

*High skilled white collar reincodieren

replace collar = 1 if (F100_isco88_3d >= 111) & (F100_isco88_3d <=348)

*Low skilled white collar reincodieren

replace collar = 2 if (F100_isco88_3d >= 411) & (F100_isco88_3d <=522)

*High skilled blue collar reincodieren

replace collar = 3 if (F100_isco88_3d >= 610) & (F100_isco88_3d <=744)

*Low skilled blue collar reincodieren

replace collar = 4 if (F100_isco88_3d >= 810) & (F100_isco88_3d <=998)

*Streitkräfte gesondert reincodieren

replace collar = 5 if (F100_isco88_3d >= 11) & (F100_isco88_3d <=11)

*Streitkräfte ausschließen

drop if collar == 5

*--> 65 gelöscht

*Unklare Berufsbezeichnung ausschließen

drop if collar == 99

*-->13 gelöscht

*Label für collar Varianten in Variable eintragen

*Missings bleiben 99

*4-Schritt-collar

label define collar 1 "high skilled white collar" 2 "low skilled white collar" 3 "high skilled blue collar" 4 "low skilled blue collar" 5 "Streitkräfte"

tab collar

tab collar, nolabel

*2-Schritt-collar

gen collar2 = 99

replace collar2 = 1 if (collar >= 1)

replace collar2 = 2 if (collar >= 3)

label define collar2 1 "white" 2 "blue"

*Dummy für Gender erstellen

gen GenderDummy = 0

replace GenderDummy = 1 if S1 == 2

*Dummy für Collar erstellen

gen CollarDummy = collar2

replace CollarDummy = 0 if collar2 == 1

replace CollarDummy = 1 if collar2 == 2

*Dummy für Kindstatus erstellen

gen Kindstatus = F1604

gen KindstatusDummy = Kindstatus

replace KindstatusDummy = 0 if Kindstatus == 1

replace KindstatusDummy = 1 if Kindstatus == 2

*Belastungen labeln

gen Kopfschmerzen = F1500_07

gen Schlafstörungen = F1500_14

gen Müdigkeit = F1500_15

gen Reizbarkeit = F1500_18

gen Niedergeschlagenheit = F1500_19

gen KörperlicheErschöpfung = F1500_21

gen EmotionaleErschöpfung = F1500_22

gen RückenKreuzschmerzen = F1500_01

gen SchulterNackenschmerzen = F1500_02

gen Hüftschmerzen = F1500_04

gen HerzschmerzenEngeStiche = F1500_08

gen HautreizungenJuckreiz = F1500_13

gen MagenVerdauungsbeschwerden = F1500_16

gen HörverschlechterungOhrgeräusche = F1500_17

gen Schwindel = F1500_20

*Vollzeit/Teilzeit Variable erstellen

gen Vollzeit35=99

replace Vollzeit35 = 1 if az>35

replace Vollzeit35 = 0 if az<36

tab Vollzeit35

label define Vollzeit35 0 "nein" 1 "ja"

*KopfschmerzenDummy erstellen für logsitische Regression später

gen KopfschmerzenDummy = Kopfschmerzen

replace KopfschmerzenDummy = 1 if Kopfschmerzen == 1

replace KopfschmerzenDummy = 0 if Kopfschmerzen == 2

*SchlafstörungenDummy erstellen für logsitische Regression später

gen SchlafstörungenDummy = Schlafstörungen

replace SchlafstörungenDummy = 1 if Schlafstörungen == 1

replace SchlafstörungenDummy = 0 if Schlafstörungen == 2

*MüdigkeitDummy erstellen für logsitische Regression später

gen MüdigkeitDummy = Schlafstörungen

replace MüdigkeitDummy = 1 if Müdigkeit == 1

replace MüdigkeitDummy = 0 if Müdigkeit == 2

*ReizbarkeitDummy erstellen für logsitische Regression später

gen ReizbarkeitDummy = Reizbarkeit

replace ReizbarkeitDummy = 1 if Reizbarkeit == 1

replace ReizbarkeitDummy = 0 if Reizbarkeit == 2

*NiedergeschlagenheitDummy erstellen für logsitische Regression später

gen NiedergeschlagenheitDummy = Niedergeschlagenheit

replace NiedergeschlagenheitDummy = 1 if Niedergeschlagenheit == 1

replace NiedergeschlagenheitDummy = 0 if Niedergeschlagenheit == 2

*KörperlicheErschöpfungDummy erstellen für logsitische Regression später

gen KörperlicheErschöpfungDummy = KörperlicheErschöpfung

replace KörperlicheErschöpfungDummy = 1 if KörperlicheErschöpfung == 1

replace KörperlicheErschöpfungDummy = 0 if KörperlicheErschöpfung == 2

*EmotionaleErschöpfungDummy erstellen für logsitische Regression später

gen EmotionaleErschöpfungDummy = EmotionaleErschöpfung

replace EmotionaleErschöpfungDummy = 1 if EmotionaleErschöpfung == 1

replace EmotionaleErschöpfungDummy = 0 if EmotionaleErschöpfung == 2

*Belastungsscore erstellen aus 0-1-Belastungsvariablen

gen Kopfschmerzen01 = 0

gen Schlafstörungen01 = 0

gen Müdigkeit01 = 0

gen Reizbarkeit01 = 0

gen Niedergeschlagenheit01 = 0

gen KörperlicheErschöpfung01 = 0

gen EmotionaleErschöpfung01 = 0

replace Kopfschmerzen01 = 1 if (F1500_07 <2)

replace Schlafstörungen01 = 1 if (F1500_14 <2)

replace Müdigkeit01 = 1 if (F1500_15 <2)

replace Reizbarkeit01 = 1 if (F1500_18 <2)

replace Niedergeschlagenheit01 = 1 if (F1500_19 <2)

replace KörperlicheErschöpfung01 = 1 if (F1500_21 <2)

replace EmotionaleErschöpfung01 = 1 if (F1500_22 <2)

gen Belastungsscore = Kopfschmerzen01+ Schlafstörungen01+ Müdigkeit01+ Reizbarkeit01+ Niedergeschlagenheit01+ KörperlicheErschöpfung01+ EmotionaleErschöpfung01

gen BelastungsscoreKurz = Kopfschmerzen01+ Schlafstörungen01+ Müdigkeit01+ Reizbarkeit01+ Niedergeschlagenheit01

gen wave = 2018

gen wave2018 = 1

*Variablen ergäenzen fürs mergen

gen Intnr = intnr

*Familienstatus

gen Familienstatus = F1600

*Eingetragene Partnerschaft zu Ehe machen

replace Familienstatus = 1 if (F1600 >=5)

*Missings

*Geschlecht

tab S1

*keine Missings bei Geschlecht, keine Löschungen

*Belastungen

tab F1500_07

tab F1500_07, nolabel

drop if F1500_07 == 9

*49 gelöscht

tab F1500_14

drop if F1500_14 == 9

*24 gelöscht

tab F1500_15

drop if F1500_15 == 9

*9 gelöscht

tab F1500_18

drop if F1500_18 == 9

*16 gelöscht

tab F1500_19

drop if F1500_19 == 9

* 17 gelöscht

tab F1500_21

drop if F1500_21 == 9

* 12 gelöscht

tab F1500_22

drop if F1500_22 == 9

*20 gelöscht

* --> 147 wegen fehlender Belastungsangaben gelöscht

*Alter:

tab zpalter

drop if zpalter == 9999

*--> 170 wegen fehlender Altersdaten gelöscht

*Arbeitszeit:

tab az

*keine Missings in Arbeitzeit, keine Löschungen

*Kindmissings raus

drop if Kindstatus == 9

save "C:\Users\julia\Desktop\MHH WiMi\Daten 2018 nach Aufbau(neu).dta"

## 4. Step 3: Merging of three datasets and analyses

**Dofile Merging, Vorbereitung und Analysen

**Jahresvergleiche Deutschland 2006, 2012, 2018

log using "C:\Users\julia\Desktop\MHH WiMi\06 Paper 2\OutputDoku.smcl"

*DS zusammenführen

use "C:\Users\julia\Desktop\MHH WiMi\06 Paper 2\Daten 2018 nach Aufbau(neu).dta"

append using "C:\Users\julia\Desktop\MHH WiMi\06 Paper 2\Daten 2012 nach Aufbau(neu).dta" "C:\Users\julia\Desktop\MHH WiMi\06 Paper 2\Daten 2006 nach Aufbau(neu).dta"

*Übergreifende Gewichtungsvariable generieren

gen GewAll=0

replace GewAll = gew3_05 if (wave == 2006)

replace GewAll = Gew2012 if (wave == 2012)

replace GewAll = gew2018 if (wave == 2018)

*Großen DS speichern

save "C:\Users\julia\Desktop\MHH WiMi\06 Paper 2\Datensatz 2006 2012 2018 (neu).dta"

*Analysen Jahresvergleiche Deutschland

use "C:\Users\julia\Desktop\MHH WiMi\06 Paper 2\Datensatz 2006 2012 2018 (neu).dta"

*FAKOTORENANALYSE

factor Kopfschmerzen Schlafstörungen Müdigkeit Reizbarkeit Niedergeschlagenheit , pcf

rotate

predict factor1

screeplot

*Alle Symptome

factor Kopfschmerzen Schlafstörungen Müdigkeit Reizbarkeit Niedergeschlagenheit RückenKreuzschmerzen SchulterNackenschmerzen Hüftschmerzen HerzschmerzenEngeStiche Schwindel HörverschlechterungOhrgeräusche HautreizungenJuckreiz MagenVerdauungsbeschwerden, pcf

rotate

screeplot

*Horn’s parallel analysis for principal component analysis

paran Kopfschmerzen Schlafstörungen Müdigkeit Reizbarkeit Niedergeschlagenheit, all graph quietly seed(1)

*McDonalds Omega

gen körperlicheerschöpfung = KörperlicheErschöpfung

gen schlafstörungen= Schlafstörungen

gen müdigkeit= Müdigkeit

gen reizbarkeit= Reizbarkeit

gen niedergeschlagenheit= Niedergeschlagenheit

gen belastungsscorekurz=BelastungsscoreKurz

sem (belastungsscorekurz -> kopfschmerzen schlafstörungen müdigkeit reizbarkeit niedergeschlagenheit)

omegacoef kopfschmerzen schlafstörungen müdigkeit reizbarkeit niedergeschlagenheit

*DESCRIPTIVE DATA

mean zpalter, over(wave)

estat sd

*Tabelle mit Durchschnittsaltern pro Welle anzeigen

collapse (mean) zpalter, by(wave)

list, sepby(wave)

table S1 wave

table collar S1 wave

tab S1 collar if wave ==2006

tab S1 collar if wave ==2012

tab S1 collar if wave ==2018

*Collarverteilungen nach Wave (Häufigkeiten %)

tab collar if wave==2006

tab collar if wave==2012

tab collar if wave==2018

*Genderverteilungen nach Wave (Häufigkeiten %)

tab S1 if wave == 2006

tab S1 if wave == 2012

tab S1 if wave == 2018

*Genderverteilungen nach Wave und Jahr (Häufigkeiten %)

tab S1 if collar==1 & wave==2006

tab S1 if collar==2 & wave==2006

tab S1 if collar==3 & wave==2006

tab S1 if collar==4 & wave==2006

tab S1 if collar==1 & wave==2012

tab S1 if collar==2 & wave==2012

tab S1 if collar==3 & wave==2012

tab S1 if collar==4 & wave==2012

tab S1 if collar==1 & wave==2018

tab S1 if collar==2 & wave==2018

tab S1 if collar==3 & wave==2018

tab S1 if collar==4 & wave==2018

*ANALYSES

*Interne Konsistenz der Skala für psychosomatische Belastungen

alpha Kopfschmerzen Niedergeschlagenheit Schlafstörungen Müdigkeit Reizbarkeit

alpha Kopfschmerzen Niedergeschlagenheit Schlafstörungen Müdigkeit Reizbarkeit, std item detail

*Mittelwerte BelastungsscoreKurz nach Jahr und S1 Overall

table S1 wave, contents(mean BelastungsscoreKurz)

table S1 wave, contents(sd BelastungsscoreKurz)

*Graph zum Verlauf der Belastung je Wave und Geschlecht zeichnen

mean BelastungsscoreKurz, over (wave S1)

quietly anova BelastungsscoreKurz S1##wave

margins wave##S1

marginsplot, noci ytitle(Mean BelastungsscoreKurz)

*Mittelwerte BelastungsscoreKurz nach Jahr und S1 nach Collar

*Each year

keep if wave==2006

table S1, contents(mean BelastungsscoreKurz)

table S1, contents(sd BelastungsscoreKurz)

clear

use "C:\Users\julia\Desktop\MHH WiMi\06 Paper 2\Datensatz 2006 2012 2018 (final).dta"

keep if wave==2012

table S1, contents(mean BelastungsscoreKurz)

table S1, contents(sd BelastungsscoreKurz)

clear

use "C:\Users\julia\Desktop\MHH WiMi\06 Paper 2\Datensatz 2006 2012 2018 (final).dta"

keep if wave==2018

table S1, contents(mean BelastungsscoreKurz)

table S1, contents(sd BelastungsscoreKurz)

clear

use "C:\Users\julia\Desktop\MHH WiMi\06 Paper 2\Datensatz 2006 2012 2018 (final).dta"

*by collar

keep if wave==2006

table collar S1, contents(mean BelastungsscoreKurz)

table collar S1, contents(sd BelastungsscoreKurz)

clear

use "C:\Users\julia\Desktop\MHH WiMi\06 Paper 2\Datensatz 2006 2012 2018 (neu).dta"

keep if wave==2012

table collar S1, contents(mean BelastungsscoreKurz)

table collar S1, contents(sd BelastungsscoreKurz)

clear

use "C:\Users\julia\Desktop\MHH WiMi\06 Paper 2\Datensatz 2006 2012 2018 (neu).dta"

keep if wave==2018

table collar S1, contents(mean BelastungsscoreKurz)

table collar S1, contents(sd BelastungsscoreKurz)

clear

use "C:\Users\julia\Desktop\MHH WiMi\06 Paper 2\Datensatz 2006 2012 2018 (neu).dta"

*Mittelwertsunterschiede testen: Signifikanzen der Genderunterschiede

*innerhalb eines Jahres/overall testen per T-Test/Bootsstrap

keep if wave==2006

bootstrap t=r(t), reps (2000): ttest BelastungsscoreKurz, by(S1)

clear

use "C:\Users\julia\Desktop\MHH WiMi\Datensatz 2006 2012 2018 (neu).dta"

keep if wave==2012

bootstrap t=r(t), reps (2000): ttest BelastungsscoreKurz, by(S1)

clear

use "C:\Users\julia\Desktop\MHH WiMi\Datensatz 2006 2012 2018 (neu).dta"

keep if wave==2018

bootstrap t=r(t), reps (2000): ttest BelastungsscoreKurz, by(S1)

clear

use "C:\Users\julia\Desktop\MHH WiMi\Datensatz 2006 2012 2018 (neu).dta"

*Mittelwertsunterschiede testen: Signifikanzen der Genderunterschiede

*innerhalb eines Jahres und eines Collars testen per T-Test/Bootstrap

keep if collar == 1

keep if wave==2006

bootstrap t=r(t), reps (2000): ttest BelastungsscoreKurz, by(S1)

clear

use "C:\Users\julia\Desktop\MHH WiMi\06 Paper 2\Datensatz 2006 2012 2018 (neu).dta"

keep if collar == 1

keep if wave==2012

bootstrap t=r(t), reps (2000): ttest BelastungsscoreKurz, by(S1)

clear

use "C:\Users\julia\Desktop\MHH WiMi\06 Paper 2\Datensatz 2006 2012 2018 (neu).dta"

keep if collar == 1

keep if wave==2018

bootstrap t=r(t), reps (2000): ttest BelastungsscoreKurz, by(S1)

clear

use "C:\Users\julia\Desktop\MHH WiMi\06 Paper 2\Datensatz 2006 2012 2018 (neu).dta"

keep if collar == 2

keep if wave==2006

bootstrap t=r(t), reps (2000): ttest BelastungsscoreKurz, by(S1)

clear

use "C:\Users\julia\Desktop\MHH WiMi\06 Paper 2\Datensatz 2006 2012 2018 (neu).dta"

keep if collar == 2

keep if wave==2012

bootstrap t=r(t), reps (2000): ttest BelastungsscoreKurz, by(S1)

clear

use "C:\Users\julia\Desktop\MHH WiMi\06 Paper 2\Datensatz 2006 2012 2018 (neu).dta"

keep if collar == 2

keep if wave==2018

bootstrap t=r(t), reps (2000): ttest BelastungsscoreKurz, by(S1)

clear

use "C:\Users\julia\Desktop\MHH WiMi\06 Paper 2\Datensatz 2006 2012 2018 (neu).dta"

keep if collar == 3

keep if wave==2006

bootstrap t=r(t), reps (2000): ttest BelastungsscoreKurz, by(S1)

clear

use "C:\Users\julia\Desktop\MHH WiMi\06 Paper 2\Datensatz 2006 2012 2018 (neu).dta"

keep if collar == 3

keep if wave==2012

bootstrap t=r(t), reps (2000): ttest BelastungsscoreKurz, by(S1)

clear

use "C:\Users\julia\Desktop\MHH WiMi\06 Paper 2\Datensatz 2006 2012 2018 (neu).dta"

keep if collar == 3

keep if wave==2018

bootstrap t=r(t), reps (2000): ttest BelastungsscoreKurz, by(S1)

clear

use "C:\Users\julia\Desktop\MHH WiMi\06 Paper 2\Datensatz 2006 2012 2018 (neu).dta"

keep if collar == 4

keep if wave==2006

bootstrap t=r(t), reps (2000): ttest BelastungsscoreKurz, by(S1)

clear

use "C:\Users\julia\Desktop\MHH WiMi\06 Paper 2\Datensatz 2006 2012 2018 (neu).dta"

keep if collar == 4

keep if wave==2012

bootstrap t=r(t), reps (2000): ttest BelastungsscoreKurz, by(S1)

clear

use "C:\Users\julia\Desktop\MHH WiMi\06 Paper 2\Datensatz 2006 2012 2018 (neu).dta"

keep if collar == 4

keep if wave==2018

bootstrap t=r(t), reps (2000): ttest BelastungsscoreKurz, by(S1)

clear

use "C:\Users\julia\Desktop\MHH WiMi\06 Paper 2\Datensatz 2006 2012 2018 (neu).dta"

*Lin Regression m/ Gewichtung und für zpalter und Arbeitszeit kontrolliert

reg c.BelastungsscoreKurz c.Vollzeit35 c.zpalter i.S1 [pweight=gew3_05] if wave==2006

reg c.BelastungsscoreKurz c.Vollzeit35 c.zpalter i.S1 [pweight=gew3_05] if collar==1 & wave==2006

reg c.BelastungsscoreKurz c.Vollzeit35 c.zpalter i.S1 [pweight=gew3_05] if collar==2 & wave==2006

reg c.BelastungsscoreKurz c.Vollzeit35 c.zpalter i.S1 [pweight=gew3_05] if collar==3 & wave==2006

reg c.BelastungsscoreKurz c.Vollzeit35 c.zpalter i.S1 [pweight=gew3_05] if collar==4 & wave==2006

reg c.BelastungsscoreKurz c.Vollzeit35 c.zpalter i.S1 [pweight=Gew2012] if wave==2012

reg c.BelastungsscoreKurz c.Vollzeit35 c.zpalter i.S1 [pweight=Gew2012] if collar==1 & wave==2012

reg c.BelastungsscoreKurz c.Vollzeit35 c.zpalter i.S1 [pweight=Gew2012] if collar==2 & wave==2012

reg c.BelastungsscoreKurz c.Vollzeit35 c.zpalter i.S1 [pweight=Gew2012] if collar==3 & wave==2012

reg c.BelastungsscoreKurz c.Vollzeit35 c.zpalter i.S1 [pweight=Gew2012] if collar==4 & wave==2012

reg c.BelastungsscoreKurz c.Vollzeit35 c.zpalter i.S1 [pweight=gew2018] if wave==2018

reg c.BelastungsscoreKurz c.Vollzeit35 c.zpalter i.S1 [pweight=gew2018] if collar==1 & wave==2018

reg c.BelastungsscoreKurz c.Vollzeit35 c.zpalter i.S1 [pweight=gew2018] if collar==2 & wave==2018

reg c.BelastungsscoreKurz c.Vollzeit35 c.zpalter i.S1 [pweight=gew2018] if collar==3 & wave==2018

reg c.BelastungsscoreKurz c.Vollzeit35 c.zpalter i.S1 [pweight=gew2018] if collar==4 & wave==2018

*Analyse ohne Interaktionseffekte (gewichtet)

reg c.BelastungsscoreKurz i.Vollzeit35 i.Kindstatus i.collar c.zpalter i.S1 i.wave [pweight=GewAll]

*Interaktionseffekte (gewichtet)

reg c.BelastungsscoreKurz i.Vollzeit35 i.Kindstatus c.zpalter i.S1##i.wave [pweight=GewAll]

*Sensitivitätsanalyse: Analysen wiederholt mit umfangreicheren Score

drop if RückenKreuzschmerzen==9

*(30 observations deleted)

drop if SchulterNackenschmerzen==9

*(10 observations deleted)

drop if Hüftschmerzen==9

*(12 observations deleted)

drop if HerzschmerzenEngeStiche==9

*(13 observations deleted)

drop if HautreizungenJuckreiz==9

*(8 observations deleted)

drop if MagenVerdauungsbeschwerden==9

*(8 observations deleted)

drop if HörverschlechterungOhrgeräusche==9

*(25 observations deleted)

drop if Schwindel==9

*(8 observations deleted)

gen RückenKreuzschmerzen01 = 0

replace RückenKreuzschmerzen01 =1 if (RückenKreuzschmerzen <2)

tab RückenKreuzschmerzen01

gen SchulterNackenschmerzen01 = 0

replace SchulterNackenschmerzen01= 1 if (SchulterNackenschmerzen <2)

gen Hüftschmerzen01 = 0

replace Hüftschmerzen01 = 1 if (Hüftschmerzen <2)

gen HerzschmerzenEngeStiche01 = 0

replace HerzschmerzenEngeStiche01= 1 if (HerzschmerzenEngeStiche <2)

gen HautreizungenJuckreiz01 =0

replace HautreizungenJuckreiz01 =1 if (HautreizungenJuckreiz <2)

gen MagenVerdauungsbeschwerden01 =0

replace MagenVerdauungsbeschwerden01 =1 if (MagenVerdauungsbeschwerden <2)

gen Hörverschlechterung01 =0

replace Hörverschlechterung01 =1 if (HörverschlechterungOhrgeräusche <2)

gen Schwindel01 =0

replace Schwindel01 = 1 if (Schwindel <2)

gen BelastungsscoreNeu = Kopfschmerzen01+ Schlafstörungen01+ Müdigkeit01+ Reizbarkeit01+ Niedergeschlagenheit01+ RückenKreuzschmerzen01+ SchulterNackenschmerzen01+ Hüftschmerzen01+ HerzschmerzenEngeStiche01+ HautreizungenJuckreiz01+ MagenVerdauungsbeschwerden01+ Hörverschlechterung01+ Schwindel01

save "C:\Users\julia\Desktop\MHH WiMi\06 Paper 2\Datensatz 2006 2012 2018 (final) Sensitivitätsanalyse langer Score.dta"

*Mittelwerte BelastungsscoreKurz nach Jahr und S1 nach Collar

keep if wave==2006

table collar S1, contents(mean BelastungsscoreNeu)

table collar S1, contents(sd BelastungsscoreNeu)

clear

use "C:\Users\julia\Desktop\MHH WiMi\06 Paper 2\Datensatz 2006 2012 2018 (neu) Sensitivitätsanalyse langer Score.dta"

keep if wave==2012

table collar S1, contents(mean BelastungsscoreNeu)

table collar S1, contents(sd BelastungsscoreNeu)

clear

use "C:\Users\julia\Desktop\MHH WiMi\06 Paper 2\Datensatz 2006 2012 2018 (neu) Sensitivitätsanalyse langer Score.dta"

keep if wave==2018

table collar S1, contents(mean BelastungsscoreNeu)

table collar S1, contents(sd BelastungsscoreNeu)

clear

use "C:\Users\julia\Desktop\MHH WiMi\06 Paper 2\Datensatz 2006 2012 2018 (neu) Sensitivitätsanalyse langer Score.dta"

*Lin Reg mit langem Score

reg c.BelastungsscoreNeu c.Vollzeit35 c.zpalter i.S1 [pweight=gew3_05] if wave==2006

reg c.BelastungsscoreNeu c.Vollzeit35 c.zpalter i.S1 [pweight=gew3_05] if collar==1 & wave==2006

reg c.BelastungsscoreNeu c.Vollzeit35 c.zpalter i.S1 [pweight=gew3_05] if collar==2 & wave==2006

reg c.BelastungsscoreNeu c.Vollzeit35 c.zpalter i.S1 [pweight=gew3_05] if collar==3 & wave==2006

reg c.BelastungsscoreNeu c.Vollzeit35 c.zpalter i.S1 [pweight=gew3_05] if collar==4 & wave==2006

reg c.BelastungsscoreNeu c.Vollzeit35 c.zpalter i.S1 [pweight=Gew2012] if wave==2012

reg c.BelastungsscoreNeu c.Vollzeit35 c.zpalter i.S1 [pweight=Gew2012] if collar==1 & wave==2012

reg c.BelastungsscoreNeu c.Vollzeit35 c.zpalter i.S1 [pweight=Gew2012] if collar==2 & wave==2012

reg c.BelastungsscoreNeu c.Vollzeit35 c.zpalter i.S1 [pweight=Gew2012] if collar==3 & wave==2012

reg c.BelastungsscoreNeu c.Vollzeit35 c.zpalter i.S1 [pweight=Gew2012] if collar==4 & wave==2012

reg c.BelastungsscoreNeu c.Vollzeit35 c.zpalter i.S1 [pweight=gew2018] if wave==2018

reg c.BelastungsscoreNeu c.Vollzeit35 c.zpalter i.S1 [pweight=gew2018] if collar==1 & wave==2018

reg c.BelastungsscoreNeu c.Vollzeit35 c.zpalter i.S1 [pweight=gew2018] if collar==2 & wave==2018

reg c.BelastungsscoreNeu c.Vollzeit35 c.zpalter i.S1 [pweight=gew2018] if collar==3 & wave==2018

reg c.BelastungsscoreNeu c.Vollzeit35 c.zpalter i.S1 [pweight=gew2018] if collar==4 & wave==2018

*Interaktionsanalyse mit langem Score

reg c.BelastungsscoreNeu i.Vollzeit35 i.Kindstatus c.zpalter i.S1##i.wave [pweight=GewAll]

reg c.BelastungsscoreNeu i.Vollzeit35 c.zpalter i.S1##i.wave [pweight=GewAll] if collar==1

reg c.BelastungsscoreNeu i.Vollzeit35 c.zpalter i.S1##i.wave [pweight=GewAll] if collar==2

reg c.BelastungsscoreNeu i.Vollzeit35 c.zpalter i.S1##i.wave [pweight=GewAll] if collar==3

reg c.BelastungsscoreNeu i.Vollzeit35 c.zpalter i.S1##i.wave [pweight=GewAll] if collar==4

*Sensitivitätsanalyse RegAna statt IE

reg c.BelastungsscoreKurz i.Vollzeit35 i.Kindstatus c.zpalter i.S1 i.wave [pweight=GewAll]

*Appendix

*Entwicklung Women working fulltime over the years

tab Vollzeit35 if wave==2006

tab Vollzeit35 if wave==2006 & S1==2

tab Vollzeit35 if wave==2012 & S1==2

tab Vollzeit35 if wave==2012 & S1==1

tab Vollzeit35 if wave==2018 & S1==1

tab Vollzeit35 if wave==2018 & S1==2
